# Crystal structure and Hirshfeld surface analysis of a new polymorph of (*E*)-2-(4-bromo­phen­yl)-1-[2,2-di­bromo-1-(3-nitro­phen­yl)ethen­yl]diazene

**DOI:** 10.1107/S2056989022007113

**Published:** 2022-07-14

**Authors:** Zeliha Atioğlu, Mehmet Akkurt, Namiq Q. Shikhaliyev, Naila A. Mammadova, Gülnara V. Babayeva, Victor N. Khrustalev, Ajaya Bhattarai

**Affiliations:** a Department of Aircraft Electrics and Electronics, School of Applied Sciences, Cappadocia University, Mustafapaşa, 50420 Ürgüp, Nevşehir, Turkey; b Department of Physics, Faculty of Sciences, Erciyes University, 38039 Kayseri, Turkey; c Organic Chemistry Department, Baku State University, Z. Khalilov str. 23, AZ 1148 Baku, Azerbaijan; d Azerbaijan State Pedagogical University, Uzeyir Hajibeyli str., 68, Baku, Azerbaijan; e Peoples’ Friendship University of Russia (RUDN University), Miklukho-Maklay St. 6, Moscow, 117198, Russian Federation, N. D. Zelinsky Institute of Organic Chemistry RAS, Leninsky Prosp. 47, Moscow, 119991, Russian Federation; f Department of Chemistry, M.M.A.M.C (Tribhuvan University) Biratnagar, Nepal; Moscow State University, Russia

**Keywords:** crystal structure, azo compounds, polymorphism, C—H⋯O inter­actions, Hirshfeld surface analysis

## Abstract

A new polymorph of the title compound, C_14_H_8_Br_3_N_3_O_2_, (form-2) was obtained in the same manner as the previously reported form-1 [Akkurt *et al.* (2022 ▸). *Acta Cryst.* E**78**, 732–736]. The structure of the new polymorph is stabilized by a C—H⋯O hydrogen bond that links mol­ecules into chains. These chains are linked by face-to-face π–π stacking inter­actions, resulting in a layered structure. Short inter-mol­ecular Br⋯O contacts and van der Waals inter­actions between the layers aid in the cohesion of the crystal packing. In the previously reported form-1, C—H⋯Br inter­actions connect mol­ecules into zigzag chains, which are linked by C—Br⋯π inter­actions into layers, whereas the van der Waals inter­actions between the layers stabilize the crystal packing of form-2. Hirshfeld mol­ecular surface analysis was used to compare the inter­molecular inter­actions of the polymorphs.

## Abstract 2

The a new polymorph of the title compound is reported in which the C—H⋯O hydrogen bonds and π-π stacking inter­actions link mol­ecules into the layers in the crystal.

## 1. Chemical context

Aromatic azo compounds provide ubiquitous motifs in organic chemistry and are widely used as indicators, organic dyes, pigments, radical reaction initiators, food additives, therapeutic agents, etc. (Zollinger 1994 ▸, 1995 ▸; Gurbanov *et al.*, 2020*a* ▸,*b* ▸). Moreover, in azo dyes the ligands play a crucial role in coordination chemistry and in the construction of functional materials, such as ionophores, self-assembled layers, catalysts, anti­microbial agents, liquid crystals and semiconductors (Ma *et al.*, 2020 ▸, 2021 ▸; Mahmudov *et al.*, 2010 ▸, 2013 ▸). Depending on the attached functional groups, the chemical and physical properties of azo dyes and their transition-metal complexes can be improved. The *azo-*to-*hydrazo* tautomerization as well as *E*/*Z* isomerization of azo dyes are key phenomena in the synthesis and design of new functional materials (Shixaliyev *et al.*, 2013 ▸, 2014 ▸). Moreover, an attachment of donor or acceptor centres of non-covalent bonds to the azo compounds can be applied as a synthetic strategy in the improvement of functional properties of their metal complexes (Mahmudov *et al.*, 2020 ▸, 2021 ▸, 2022 ▸). Thus, we have attached bromine and nitro substituents to the aryl rings leading to a new azo dye, (*E*)-1-(2,2-di­bromo-1-(3-nitro­phen­yl)vin­yl)-2-(4-bromo­phen­yl)di­azene, which can participate in inter­molecular halogen and hydrogen bonds as well as in π-inter­actions. 
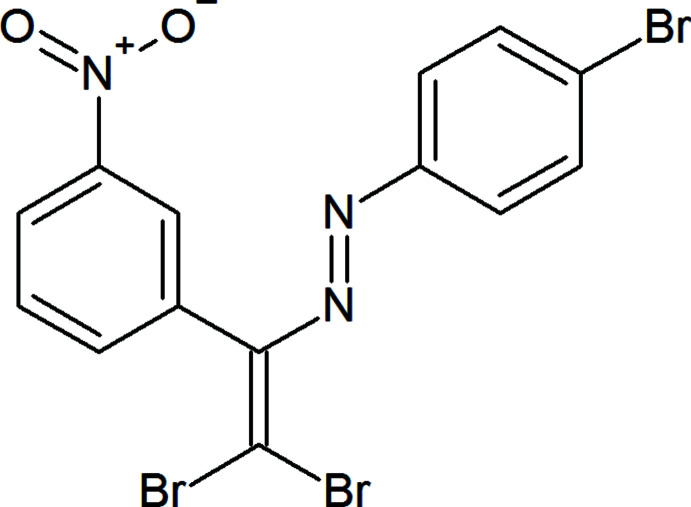


## 2. Structural commentary

**Figure 1:**
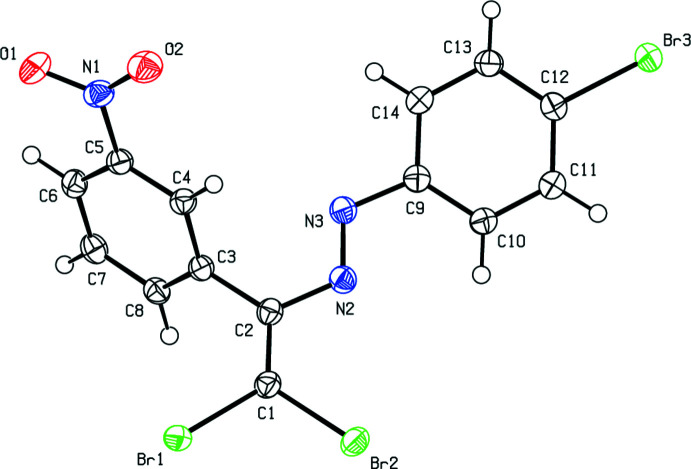
The mol­ecular structure of the title compound. Displacement ellipsoids are drawn at the 50% probability level.

A view of the mol­ecule of the new polymorph (henceforth referred to as form-2) is shown in Fig. 1 ▸. The central fragment of the mol­ecule, C1/C2/N2/N3/C3/C9/Br1/Br2, is almost planar with the largest deviation from mean plane being 0.101 (1) Å for Br1. This plane forms dihedral angles of 13.51 (7) and 61.26 (7)° with the planes of the bromine- and nitro-substituted aromatic rings, respectively. In the previously reported polymorph (form-1), the corresponding angles were 26.35 (15) and 72.57 (14)° (Akkurt *et al.*, 2022 ▸). All bond lengths and angles in the title compound are in agreement with those reported for the related azo compounds discussed in the *Database survey* section.

## 3. Supra­molecular features and Hirshfeld surface analysis

**Figure 2:**
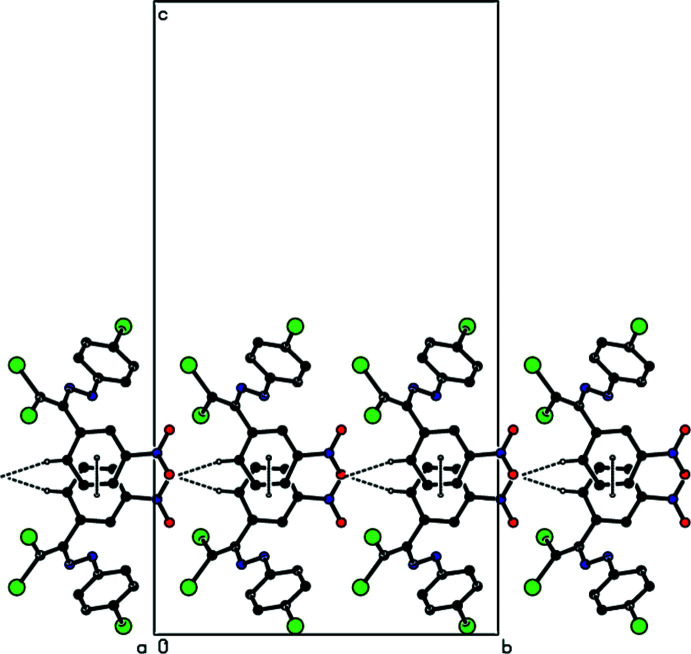
View down the *a*-axis of the C—H⋯O and π–π inter­actions (dashed lines) in the title compound.

**Figure 4:**
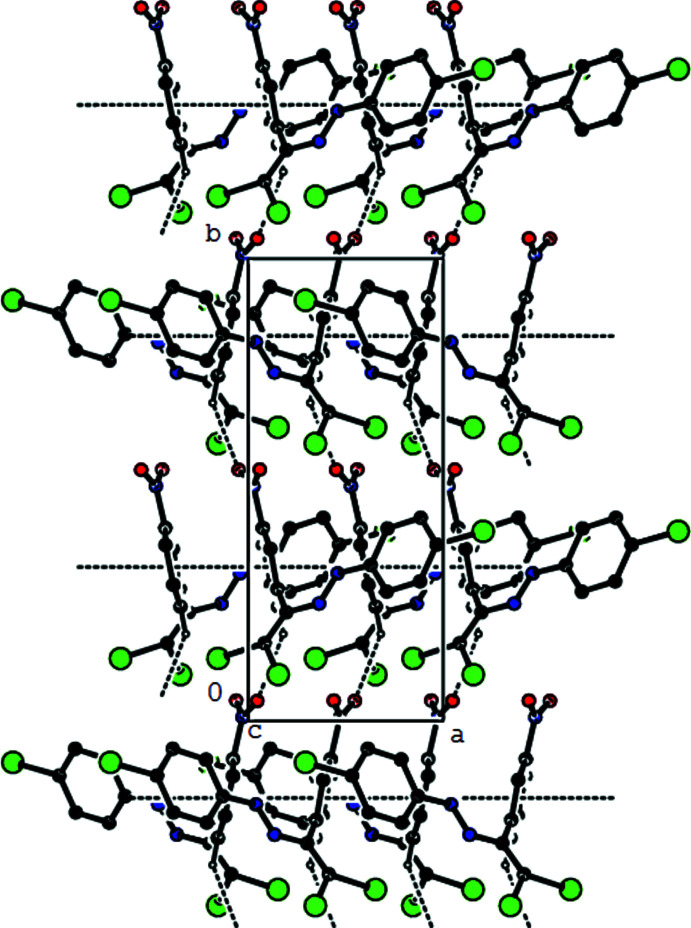
View down the *c*-axis of the C—H⋯O and π–π inter­actions (dashed lines) in the title compound.

The crystal packing of the new polymorph is stabilized by a C—H⋯O hydrogen bond that links mol­ecules into chains along the *b*-axis direction (Table 1 ▸, Figs. 2 ▸–4 ▸ ▸). These chains are joined by zigzag face-to-face π–π stacking inter­actions along the [100] direction [*Cg*1⋯*Cg*1(− 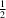 + *x*, *y*, 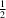 − *z*) = 3.7305 (11) Å, slippage: 2.057 Å; *Cg*1⋯*Cg*1( 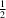 + *x*, *y*, 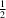 − *z*) = 3.7305 (11) Å, slippage: 0.9775 Å; where *Cg*1 is the centroid of the nitro­phenyl ring], resulting in the layers parallel to (001) (Fig. 4 ▸). Short inter-mol­ecular Br1⋯O2 contacts (Table 2 ▸) and van der Waals inter­actions between the layers help to keep the crystal packing together. In the previously reported form-1 of the title compound (Akkurt *et al.*, 2022 ▸), C—H⋯Br inter­actions connect mol­ecules, generating zigzag *C*(8) chains along the [100] direction, which are linked by C—Br⋯π inter­actions into layers parallel to (001), and van der Waals inter­actions between layers contribute to the crystal cohesion.

**Figure 5:**
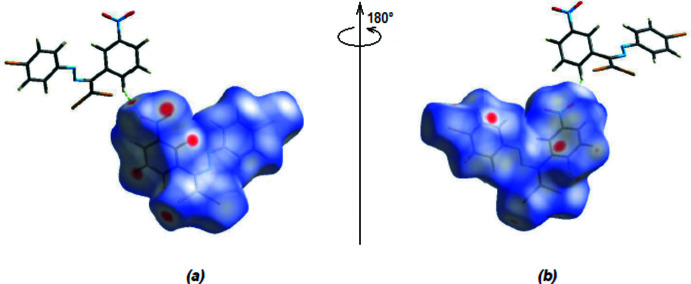
(*a*) Front and (*b*) back views of the three-dimensional Hirshfeld surface of the title compound plotted over *d*_norm_ in the range −0.1471 to 1.1715 a.u.

*Crystal Explorer 17.5* (Turner *et al.*, 2017 ▸) was used to perform a Hirshfeld surface analysis of form-2 and to generate the related two-dimensional fingerprint plots, with a standard resolution of the three-dimensional *d*_norm_ surfaces plotted over a fixed colour scale of −0.1471 (red) to +1.1715 (blue) a.u. (Fig. 5 ▸). The red areas on the surface present short contacts and negative *d*_norm_ values, which correspond to the C—H⋯O hydrogen bonds mentioned above (Table 1 ▸). The red patch that appears around O1 is due to the C8—H8⋯O1 inter­action, which is critical for the mol­ecular packing of the title compound. In form-1, the C—H⋯Br inter­actions are also prominent (Akkurt *et al.*, 2022 ▸).

**Figure 6:**
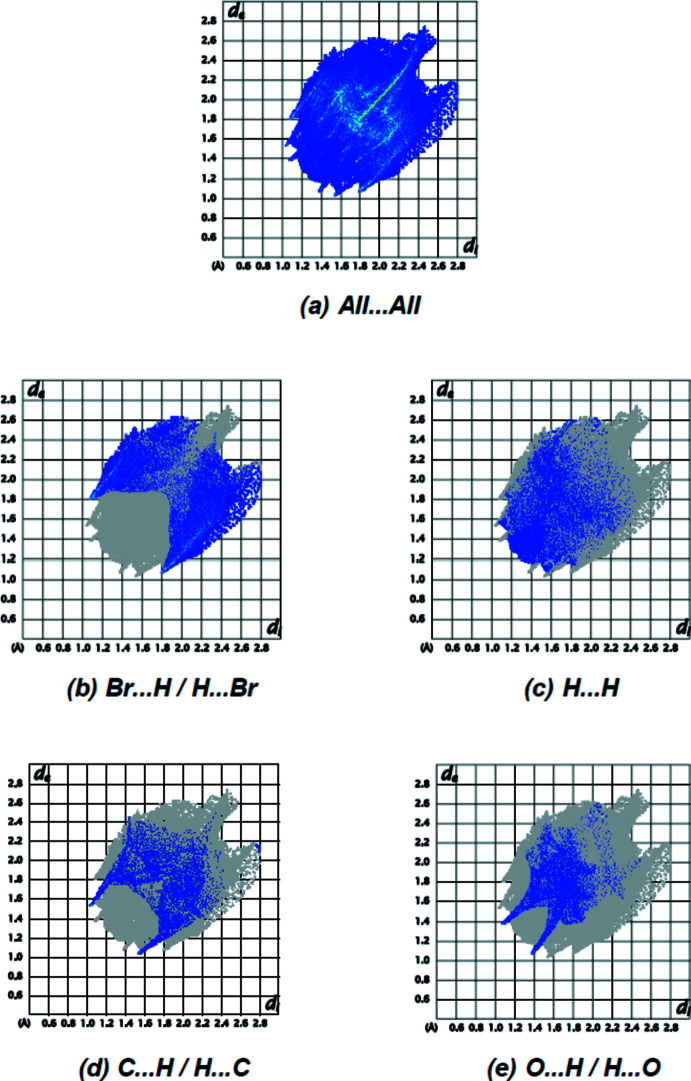
The full two-dimensional fingerprint plots for the title compound, showing (*a*) all inter­actions, and delineated into (*b*) Br⋯H/H⋯Br, (*c*) H⋯H, (*d*) C⋯H/H⋯C, and (*e*) O⋯H/H⋯O inter­actions. The *d*_i_ and *d*_e_ values are the closest inter­nal and external distances (in Å) from given points on the Hirshfeld surface.

The overall two-dimensional fingerprint plot for form-2 is given in Fig. 6 ▸ *a*, and those delineated into Br⋯H/H⋯Br (26.5%), H⋯H (12.8%), C⋯H/H⋯C (11.5%) and O⋯H/H⋯O (10.6%) contacts are shown in Fig. 6 ▸ *b*–*e*, while the numerical details for the shortest contacts are given in Table 2 ▸. Other contacts, such as Br⋯C/C⋯Br (7.7%), C⋯C (6.0%), Br⋯Br (5.8%), Br⋯O/O⋯Br (5.3%), N⋯H/H⋯N (5.3%), O⋯C/C⋯O (2.5%), Br⋯N/N⋯Br (2.3%), O⋯N/N⋯O (1.7%), O⋯O (1.3%) and N⋯C/C⋯N (0.8%), have little influence on the mol­ecular packing. For form-1, the set includes only four types of inter­actions, *viz*. Br⋯H/H⋯Br, H⋯H, C⋯H/H⋯C and O⋯H/H⋯O contacts (Akkurt *et al.*, 2022 ▸). The predominant inter­actions in both cases are Br⋯H/H⋯Br and H⋯H, constituting 26.5% and 12.8%, respectively, in form-2 *vs* 20.9% and 15.2% in form*-*1.

## 4. Database survey

A search of the Cambridge Structural Database (CSD, Version 5.42, update of September 2021; Groom *et al.*, 2016 ▸) for similar structures with the (*E*)-1-(2,2-di­bromo)-2-(4-bromo­phen­yl)diazene unit showed that the ten closest are those of CSD refcodes HEHKEO (**I**) (Akkurt *et al.*, 2022 ▸), TAZDIL (**II**) (Atioğlu *et al.*, 2022 ▸), PAXDOL (**III**) (Çelikesir *et al.*, 2022 ▸), GUPHIL (**IV**) (Özkaraca *et al.*, 2020*b* ▸), HONBUK (**V**) (Akkurt *et al.*, 2019 ▸), HONBOE (**VI**) (Akkurt *et al.*, 2019 ▸), HODQAV (**VII**) (Shikhaliyev *et al.*, 2019 ▸), XIZREG (**VIII**) (Atioğlu *et al.*, 2019 ▸), LEQXOX (**IX**) (Shikhaliyev *et al.*, 2018 ▸) and LEQXIR (**X**) (Shikhaliyev *et al.*, 2018 ▸).

C—H⋯Br inter­actions connect the mol­ecules in the crystal of the form-1 polymorph of the title compound, (**I**), resulting in zigzag *C*(8) chains along the [100] direction. These chains are connected by C—Br⋯π inter­actions into layers parallel to (001). van der Waals inter­actions between the layers contribute to the crystal cohesion.

The mol­ecules in (**II**) are joined into layers parallel to (011) by C—H⋯O and C—H⋯F hydrogen bonds. C—Br⋯π and C—F⋯π contacts, as well as π–π stacking inter­actions, strengthen the crystal packing

The mol­ecules in the crystal of (**III**) are connected into chains running parallel to [001] by C—H⋯O hydrogen bonds. C—F⋯π contacts and π–π stacking inter­actions help to consolidate the crystal packing, and short Br⋯O [2.9828 (13) Å] distances are also observed.

In the crystal of (**IV**), the mol­ecules are linked into inversion dimers *via* short halogen–halogen contacts [Cl1⋯Cl1 = 3.3763 (9) Å, C16—Cl1⋯Cl1 = 141.47 (7)° compared to the van der Waals radii sum of 3.50 Å for two chlorine atoms]. No other directional contacts could be identified, and the shortest aromatic ring-centroid separation is greater than 5.25 Å.

In the crystals of (**V**) and (**VI**), the mol­ecules are linked through weak *X*⋯Cl contacts [*X* = Cl for (**V**) and Br for (**VI**)], C—H⋯Cl and C—Cl⋯π inter­actions into sheets lying parallel to (001).

In the crystal of (**VII**), the mol­ecules are stacked in columns along [100] *via* weak C—H⋯Cl hydrogen bonds and face-to-face π–π stacking inter­actions. The crystal packing is further consolidated by short Cl⋯Cl contacts.

In (**VIII**), mol­ecules are linked by C—H⋯O hydrogen bonds into zigzag chains running parallel to [001]. The crystal packing also features C—Cl⋯π, C—F⋯π and N—O⋯π inter­actions.

In (**IX**), C—H⋯N and short Cl⋯Cl contacts are observed, and in (**X**), C—H⋯N and C—H⋯O hydrogen bonds and short Cl⋯O contacts occur.

## 5. Synthesis and crystallization

This dye was synthesized according to the reported method (Akkurt *et al.*, 2019 ▸; Maharramov *et al.*, 2018 ▸; Özkaraca *et al.*, 2020*a* ▸,*b* ▸). A 20 mL screw-neck vial was charged with DMSO (10 mL), (*E*)-1-(4-bromo­phen­yl)-2-(3-nitro­benzyl­idene)hydrazine (1 mmol), tetra­methyl­ethylenedi­amine (TMEDA; 295 mg, 2.5 mmol), CuCl (2 mg, 0.02 mmol) and CBr_4_ (4.5 mmol). After 1–3 h (until TLC analysis showed complete consumption of corresponding Schiff base), the reaction mixture was poured into a 0.01 *M* solution of HCl (100 mL, pH = 2–3), and extracted with di­chloro­methane (3 × 20 mL). The combined organic phase was washed with water (3 × 50 mL), brine (30 mL), dried over anhydrous Na_2_SO_4_ and concentrated *in vacuo* using a rotary evaporator. The residue was purified by column chromatography on silica gel using appropriate mixtures of hexane and di­chloro­methane (3/1–1/1). Crystals suitable for X-ray analysis were obtained by slow evaporation of an ethanol solution. Red solid (62%); m.p. 391 K. Analysis calculated for C_14_H_8_Br_3_N_3_O_2_ (*M* = 489.95): C 34.32, H 1.65, N 8.58; found: C 34.29, H 1.66, N 8.55%. ^1^H NMR (300 MHz, CDCl_3_) *δ* 7.90–7.44 (8H, Ar–H). ^13^C NMR (75MHz, CDCl_3_) *δ* 150.88, 148.57, 148.12, 132.81, 132.47, 132.25, 130.04, 126.40, 125.30, 124.53, 123.57, 94.10. ESI-MS: *m*/*z*: 490.91 [*M* + H]^+^.

## 6. Refinement

Crystal data, data collection and structure refinement details are summarized in Table 3 ▸. All H atoms were positioned geometrically and allowed to ride on their parent atoms (C—H = 0.95 Å) with *U*_iso_(H) = 1.2*U*_eq_(C).

## Supplementary Material

Crystal structure: contains datablock(s) I. DOI: 10.1107/S2056989022007113/yk2172sup1.cif

Structure factors: contains datablock(s) I. DOI: 10.1107/S2056989022007113/yk2172Isup2.hkl

Supporting information file. DOI: 10.1107/S2056989022007113/yk2172Isup3.cml

CCDC reference: 2189348

Additional supporting information:  crystallographic information; 3D view; checkCIF report

## Figures and Tables

**Figure 3:**
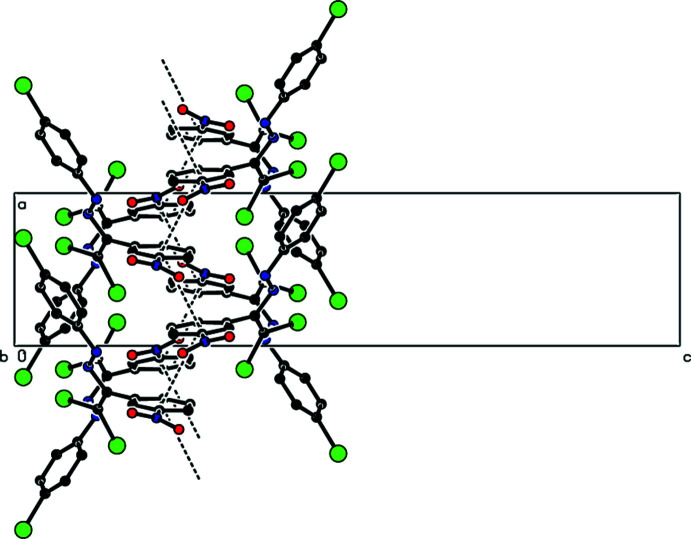
View down the *b*-axis of the C—H⋯O and π–π inter­actions (dashed lines) in the title compound.

**Table 1:** Hydrogen-bond geometry (Å, °)

*D*—H⋯*A*	*D*—H	H⋯*A*	*D*⋯*A*	*D*—H⋯*A*
C8—H8⋯O1^i^	0.95	2.56	3.394 (2)	146

Symmetry code: (i) 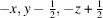 .

**Table 2:** Summary of short inter­atomic contacts (Å) in the title compound

H4⋯C13	2.67	−1 + *x*, *y*, *z*
H8⋯O1	2.56	−*x*, − 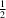 + *y*, 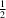 − *z*
H7⋯N3	2.78	− 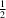 + *x*, *y*, 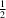 − *z*
Br1⋯O2	3.137 (2)	− 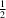 − *x*, − 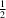 + *y*, *z*
Br1⋯H14	2.98	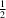 − *x*, − 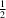 + *y*, *z*
Br2⋯H13	3.15	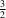 − *x*, − 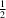 + *y*, *z*
Br3⋯H10	3.02	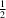 + *x*, 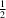 − *y*, 1 − *z*
C13⋯Br3	3.569 (2)	2 − *x*, 1 − *y*, 1 − *z*

**Table 3:** Experimental details

Crystal data	
Chemical formula	C_14_H_8_Br_3_N_3_O_2_
*M*_r_	489.96
Crystal system, space group	Orthorhombic, *P* *b* *c* *a*
Temperature (K)	100
*a*, *b*, *c* (Å)	6.6579 (1), 15.7683 (3), 29.0301 (6)
*V* (Å^3^)	3047.69 (10)
*Z*	8
Radiation type	Mo *K*α
μ (mm^−1^)	7.95
Crystal size (mm)	0.34 × 0.06 × 0.05

Data collection	
Diffractometer	Bruker AXS D8 QUEST, Photon III detector
Absorption correction	Multi-scan (*SADABS*; Krause *et al.*, 2015 ▸).
*T*_min_, *T*_max_	0.020, 0.058
No. of measured, independent and observed [*I* > 2σ(*I*)] reflections	67179, 5538, 4620
*R*_int_	0.033
(sin θ/λ)_max_ (Å^−1^)	0.758

Refinement	
*R*[*F* ^2^ > 2σ(*F* ^2^)], *wR*(*F* ^2^), *S*	0.024, 0.064, 1.05
No. of reflections	5538
No. of parameters	199
H-atom treatment	H-atom parameters constrained
Δρ_max_, Δρ_min_ (e Å^−3^)	0.68, −0.52

Computer programs: *APEX3* and *SAINT* (Bruker, 2018 ▸), *SHELXT* (Sheldrick, 2015*a* ▸), *SHELXL2018* (Sheldrick, 2015*b* ▸), *ORTEP-3 for Windows* (Farrugia, 2012 ▸) and *PLATON* (Spek, 2020 ▸).

## supplementary crystallographic information

### Crystal data

**Table:**

C_14_H_8_Br_3_N_3_O_2_	*D*_x_ = 2.136 Mg m^−^^3^
*M**_r_* = 489.96	Mo *K*α radiation, λ = 0.71073 Å
Orthorhombic, *P**b**c**a*	Cell parameters from 9783 reflections
*a* = 6.6579 (1) Å	θ = 2.7–34.8°
*b* = 15.7683 (3) Å	µ = 7.95 mm^−^^1^
*c* = 29.0301 (6) Å	*T* = 100 K
*V* = 3047.69 (10) Å^3^	Needle, red
*Z* = 8	0.34 × 0.06 × 0.05 mm
*F*(000) = 1872	

### Data collection

**Table:**

Bruker AXS D8 QUEST, Photon III detector diffractometer	5538 independent reflections
Radiation source: fine-focus sealed X-Ray tube	4620 reflections with *I* > 2σ(*I*)
Graphite monochromator	*R*_int_ = 0.033
Detector resolution: 7.31 pixels mm^-1^	θ_max_ = 32.6°, θ_min_ = 2.6°
φ and ω shutterless scans	*h* = −10→10
Absorption correction: multi-scan (SADABS; Krause *et al.*, 2015).	*k* = −23→23
*T*_min_ = 0.020, *T*_max_ = 0.058	*l* = −43→43
67179 measured reflections	

### Refinement

**Table:**

Refinement on *F*^2^	Primary atom site location: structure-invariant direct methods
Least-squares matrix: full	Secondary atom site location: difference Fourier map
*R*[*F*^2^ > 2σ(*F*^2^)] = 0.024	Hydrogen site location: inferred from neighbouring sites
*wR*(*F*^2^) = 0.064	H-atom parameters constrained
*S* = 1.05	*w* = 1/[σ^2^(*F*_o_^2^) + (0.0318*P*)^2^ + 2.0035*P*] where *P* = (*F*_o_^2^ + 2*F*_c_^2^)/3
5538 reflections	(Δ/σ)_max_ = 0.002
199 parameters	Δρ_max_ = 0.68 e Å^−^^3^
0 restraints	Δρ_min_ = −0.52 e Å^−^^3^

### Special details

**Table:**

Geometry. All esds (except the esd in the dihedral angle between two l.s. planes) are estimated using the full covariance matrix. The cell esds are taken into account individually in the estimation of esds in distances, angles and torsion angles; correlations between esds in cell parameters are only used when they are defined by crystal symmetry. An approximate (isotropic) treatment of cell esds is used for estimating esds involving l.s. planes.
Refinement. Refinement of F^2^ against ALL reflections. The weighted R-factor wR and goodness of fit S are based on F^2^, conventional R-factors R are based on F, with F set to zero for negative F^2^. The threshold expression of F^2^ > 2sigma(F^2^) is used only for calculating R-factors(gt) etc. and is not relevant to the choice of reflections for refinement. R-factors based on F^2^ are statistically about twice as large as those based on F, and R- factors based on ALL data will be even larger.

### Fractional atomic coordinates and isotropic or equivalent isotropic displacement parameters (Å^2^)

**Table:**

	*x*	*y*	*z*	*U*_iso_*/*U*_eq_	
Br1	−0.15339 (3)	0.13530 (2)	0.34586 (2)	0.02722 (5)	
Br2	0.15481 (3)	0.10023 (2)	0.42549 (2)	0.02936 (5)	
Br3	1.20530 (3)	0.41027 (2)	0.48664 (2)	0.02853 (5)	
O1	−0.0475 (3)	0.54340 (9)	0.25282 (6)	0.0379 (3)	
O2	0.0602 (3)	0.54406 (9)	0.32311 (5)	0.0353 (3)	
N1	0.0276 (2)	0.50824 (10)	0.28639 (6)	0.0272 (3)	
N2	0.3641 (2)	0.25393 (10)	0.38915 (6)	0.0238 (3)	
N3	0.4595 (2)	0.31953 (10)	0.37700 (5)	0.0237 (3)	
C1	0.0855 (3)	0.16829 (11)	0.37477 (6)	0.0238 (3)	
C2	0.1967 (3)	0.23522 (11)	0.36103 (6)	0.0226 (3)	
C3	0.1509 (2)	0.28572 (11)	0.31884 (6)	0.0215 (3)	
C4	0.1146 (3)	0.37242 (11)	0.32230 (6)	0.0221 (3)	
H4	0.115991	0.400016	0.351386	0.027*	
C5	0.0763 (3)	0.41751 (11)	0.28225 (6)	0.0224 (3)	
C6	0.0770 (3)	0.38058 (12)	0.23897 (6)	0.0236 (3)	
H6	0.050338	0.413167	0.212131	0.028*	
C7	0.1178 (3)	0.29485 (13)	0.23606 (6)	0.0250 (3)	
H7	0.121139	0.268071	0.206755	0.030*	
C8	0.1540 (3)	0.24729 (11)	0.27555 (6)	0.0228 (3)	
H8	0.180911	0.188307	0.273038	0.027*	
C9	0.6294 (3)	0.33715 (12)	0.40511 (6)	0.0233 (3)	
C10	0.7117 (3)	0.27990 (12)	0.43672 (7)	0.0254 (3)	
H10	0.650813	0.226090	0.441376	0.030*	
C11	0.8820 (3)	0.30204 (12)	0.46115 (7)	0.0267 (3)	
H11	0.940184	0.263540	0.482488	0.032*	
C12	0.9671 (3)	0.38174 (12)	0.45398 (6)	0.0239 (3)	
C13	0.8856 (3)	0.43973 (12)	0.42367 (6)	0.0257 (3)	
H13	0.944333	0.494183	0.419849	0.031*	
C14	0.7154 (3)	0.41659 (12)	0.39883 (6)	0.0248 (3)	
H14	0.657723	0.455267	0.377500	0.030*	

### Atomic displacement parameters (Å^2^)

**Table:**

	*U*^11^	*U*^22^	*U*^33^	*U*^12^	*U*^13^	*U*^23^
Br1	0.02466 (8)	0.02296 (8)	0.03404 (10)	−0.00303 (6)	−0.00384 (7)	0.00397 (7)
Br2	0.02669 (9)	0.02915 (9)	0.03225 (10)	0.00022 (7)	−0.00123 (7)	0.01012 (7)
Br3	0.02656 (9)	0.02931 (9)	0.02972 (9)	−0.00397 (7)	−0.00470 (7)	0.00276 (7)
O1	0.0417 (9)	0.0284 (7)	0.0437 (9)	0.0056 (6)	−0.0074 (7)	0.0110 (7)
O2	0.0422 (8)	0.0260 (7)	0.0378 (8)	0.0048 (6)	0.0005 (7)	−0.0047 (6)
N1	0.0234 (7)	0.0231 (7)	0.0352 (8)	0.0012 (6)	0.0018 (6)	0.0035 (6)
N2	0.0218 (6)	0.0251 (7)	0.0246 (7)	−0.0002 (5)	0.0002 (6)	0.0006 (6)
N3	0.0220 (6)	0.0243 (7)	0.0248 (7)	0.0003 (5)	−0.0002 (5)	0.0003 (6)
C1	0.0222 (7)	0.0227 (7)	0.0264 (8)	0.0017 (6)	−0.0009 (6)	0.0028 (6)
C2	0.0223 (7)	0.0222 (7)	0.0232 (8)	0.0019 (6)	−0.0003 (6)	0.0003 (6)
C3	0.0188 (7)	0.0219 (7)	0.0237 (8)	0.0000 (6)	0.0000 (6)	0.0010 (6)
C4	0.0212 (7)	0.0215 (7)	0.0237 (8)	0.0007 (6)	0.0006 (6)	0.0006 (6)
C5	0.0185 (7)	0.0214 (7)	0.0274 (8)	0.0001 (6)	0.0003 (6)	0.0016 (6)
C6	0.0177 (7)	0.0290 (8)	0.0242 (8)	−0.0007 (6)	0.0004 (6)	0.0036 (6)
C7	0.0210 (7)	0.0307 (9)	0.0232 (8)	−0.0013 (6)	0.0006 (6)	−0.0031 (7)
C8	0.0200 (7)	0.0223 (7)	0.0262 (8)	−0.0001 (6)	−0.0013 (6)	−0.0026 (6)
C9	0.0235 (7)	0.0235 (8)	0.0230 (8)	0.0007 (6)	0.0002 (6)	−0.0008 (6)
C10	0.0245 (8)	0.0241 (8)	0.0275 (8)	−0.0015 (6)	−0.0015 (7)	0.0032 (7)
C11	0.0274 (8)	0.0252 (8)	0.0275 (9)	0.0002 (7)	−0.0035 (7)	0.0030 (7)
C12	0.0225 (7)	0.0263 (8)	0.0227 (8)	−0.0011 (6)	−0.0006 (6)	−0.0012 (6)
C13	0.0281 (8)	0.0232 (8)	0.0259 (8)	−0.0024 (7)	−0.0004 (7)	0.0004 (6)
C14	0.0266 (8)	0.0235 (8)	0.0244 (8)	0.0014 (6)	−0.0004 (6)	0.0028 (6)

### Geometric parameters (Å, º)

**Table:**

Br1—C1	1.8723 (18)	C6—C7	1.381 (3)
Br2—C1	1.8796 (18)	C6—H6	0.9500
Br3—C12	1.9016 (18)	C7—C8	1.391 (3)
O1—N1	1.227 (2)	C7—H7	0.9500
O2—N1	1.226 (2)	C8—H8	0.9500
N1—C5	1.472 (2)	C9—C14	1.389 (3)
N2—N3	1.264 (2)	C9—C10	1.399 (3)
N2—C2	1.413 (2)	C10—C11	1.382 (3)
N3—C9	1.422 (2)	C10—H10	0.9500
C1—C2	1.349 (2)	C11—C12	1.394 (3)
C2—C3	1.493 (2)	C11—H11	0.9500
C3—C4	1.392 (2)	C12—C13	1.380 (3)
C3—C8	1.395 (3)	C13—C14	1.392 (3)
C4—C5	1.386 (2)	C13—H13	0.9500
C4—H4	0.9500	C14—H14	0.9500
C5—C6	1.385 (3)		
			
O2—N1—O1	123.67 (17)	C6—C7—H7	119.6
O2—N1—C5	118.68 (16)	C8—C7—H7	119.6
O1—N1—C5	117.64 (17)	C7—C8—C3	120.37 (17)
N3—N2—C2	113.96 (15)	C7—C8—H8	119.8
N2—N3—C9	113.53 (15)	C3—C8—H8	119.8
C2—C1—Br1	123.43 (14)	C14—C9—C10	120.46 (17)
C2—C1—Br2	122.92 (14)	C14—C9—N3	115.35 (16)
Br1—C1—Br2	113.64 (9)	C10—C9—N3	124.18 (17)
C1—C2—N2	115.18 (16)	C11—C10—C9	119.65 (17)
C1—C2—C3	123.21 (16)	C11—C10—H10	120.2
N2—C2—C3	121.60 (15)	C9—C10—H10	120.2
C4—C3—C8	119.61 (16)	C10—C11—C12	118.98 (17)
C4—C3—C2	120.02 (16)	C10—C11—H11	120.5
C8—C3—C2	120.30 (16)	C12—C11—H11	120.5
C5—C4—C3	118.36 (17)	C13—C12—C11	122.18 (17)
C5—C4—H4	120.8	C13—C12—Br3	119.28 (14)
C3—C4—H4	120.8	C11—C12—Br3	118.53 (14)
C6—C5—C4	122.99 (17)	C12—C13—C14	118.48 (17)
C6—C5—N1	118.92 (16)	C12—C13—H13	120.8
C4—C5—N1	118.07 (16)	C14—C13—H13	120.8
C7—C6—C5	117.88 (17)	C9—C14—C13	120.23 (17)
C7—C6—H6	121.1	C9—C14—H14	119.9
C5—C6—H6	121.1	C13—C14—H14	119.9
C6—C7—C8	120.75 (17)		
			
C2—N2—N3—C9	−179.14 (15)	C4—C5—C6—C7	0.1 (3)
Br1—C1—C2—N2	175.57 (13)	N1—C5—C6—C7	−178.28 (16)
Br2—C1—C2—N2	−3.5 (2)	C5—C6—C7—C8	0.9 (3)
Br1—C1—C2—C3	−5.5 (3)	C6—C7—C8—C3	−0.4 (3)
Br2—C1—C2—C3	175.44 (13)	C4—C3—C8—C7	−1.0 (3)
N3—N2—C2—C1	−176.40 (16)	C2—C3—C8—C7	−177.89 (16)
N3—N2—C2—C3	4.6 (2)	N2—N3—C9—C14	−168.04 (16)
C1—C2—C3—C4	121.4 (2)	N2—N3—C9—C10	13.1 (3)
N2—C2—C3—C4	−59.7 (2)	C14—C9—C10—C11	−1.3 (3)
C1—C2—C3—C8	−61.7 (2)	N3—C9—C10—C11	177.51 (18)
N2—C2—C3—C8	117.13 (19)	C9—C10—C11—C12	0.6 (3)
C8—C3—C4—C5	1.9 (2)	C10—C11—C12—C13	0.8 (3)
C2—C3—C4—C5	178.81 (16)	C10—C11—C12—Br3	−178.42 (15)
C3—C4—C5—C6	−1.5 (3)	C11—C12—C13—C14	−1.5 (3)
C3—C4—C5—N1	176.87 (15)	Br3—C12—C13—C14	177.71 (14)
O2—N1—C5—C6	−167.63 (17)	C10—C9—C14—C13	0.6 (3)
O1—N1—C5—C6	13.8 (2)	N3—C9—C14—C13	−178.32 (17)
O2—N1—C5—C4	13.9 (2)	C12—C13—C14—C9	0.8 (3)
O1—N1—C5—C4	−164.68 (17)		

### Hydrogen-bond geometry (Å, º)

**Table:**

*D*—H···*A*	*D*—H	H···*A*	*D*···*A*	*D*—H···*A*
C8—H8···O1^i^	0.95	2.56	3.394 (2)	146

Symmetry code: (i) −*x*, *y*−1/2, −*z*+1/2.
